# The Role of Bacteria in Central Nervous System Tumors: Opportunities and Challenges

**DOI:** 10.3390/microorganisms12061053

**Published:** 2024-05-23

**Authors:** Rui Zhang, Xueying Li, Si Zhang

**Affiliations:** Department of Neurosurgery, West China Hospital, Sichuan University, Chengdu 610041, China; 2022224020101@stu.scu.edu.cn (R.Z.); 2022224025372@stu.scu.edu.cn (X.L.)

**Keywords:** central nervous system tumor, bacteria, tumor microenvironment, targeted delivery, intestinal flora

## Abstract

Tumors of the central nervous system (CNS) are severe and refractory diseases with poor prognosis, especially for patients with malignant glioblastoma and brain metastases. Currently, numerous studies have explored the potential role of bacteria and intestinal flora in tumor development and treatment. Bacteria can penetrate the blood–brain barrier (BBB), targeting the hypoxic microenvironment at the core of tumors, thereby eliminating tumors and activating both the innate and adaptive immune responses, rendering them promising therapeutic agents for CNS tumors. In addition, engineered bacteria and derivatives, such as bacterial membrane proteins and bacterial spores, can also be used as good candidate carriers for targeted drug delivery. Moreover, the intestinal flora can regulate CNS tumor metabolism and influence the immune microenvironment through the “gut–brain axis”. Therefore, bacterial anti-tumor therapy, engineered bacterial targeted drug delivery, and intervention of the intestinal flora provide therapeutic modalities for the treatment of CNS tumors. In this paper, we performed a comprehensive review of the mechanisms and therapeutic practices of bacterial therapy for CNS tumors and discussed potential future research directions in this field.

## 1. Introduction

Tumors of the central nervous system (CNS) are a major cause of cancer-related mortality, affecting approximately 23 per 100,000 people [1]. Currently, brain tumors are primarily treated with surgery followed by chemoradiotherapy. However, the prognosis remains unfavorable with a high recurrence rate, especially for malignant tumors such as glioblastoma (GBM) [1]. While recent advances in targeted therapies, such as anti-EGFR and CAR-T, have shown promise for patients with brain tumors, the effectiveness of these treatments is often hindered by the challenges of penetrating physical barriers and managing immune-related adverse effects [2].

In 1813, Vautier et al. documented an unexpected phenomenon: the atypical tumor regression in cancer patients afflicted with gas gangrene due to *Clostridium perfringens* infection [3]. In 1866, W. Busch et al. observed that cancer patients experiencing erysipelas, a streptococcal skin infection, occasionally exhibited tumor regression. His pioneering efforts led to the first recorded successful application of *Streptococcus pyogenes* as a bacterial preparation for tumor therapy [4]. In 1891, William B. Coley noted an analogous occurrence in malignant sarcomas, prompting the development of a therapeutic bacterial vaccine known as Coley’s toxin. This concoction, comprising heat-inactivated *streptococcal* and *Serratia marcescens organisms*, demonstrated preliminary yet promising efficacy in the management of sarcomas and lymphomas during that era [5]. However, a period of dormancy occurred due to the elusive nature of its mechanism and safety concerns. In 1976, bacterial therapy in cancer treatment resurged when researchers explored the use of live bacterial agents. This was exemplified by the application of the *bacille Calmette-Guérin* vaccine as a therapeutic strategy for bladder cancer, harnessing the potential of *Mycobacterium bovis* in tumor management [6,7]. Since then, an expanding corpus of research has compellingly demonstrated the therapeutic potential of bacterial interventions in combating neoplastic diseases. Notably, this promising trend extends to the management of CNS malignancies, challenging conventional paradigms and opening new avenues for cancer therapy [8]. Utilizing gene editing technologies and the fundamentals of tumor immunology, researchers have evaluated the therapeutic potential of live bacteria for the management of CNS tumors, with a particular focus on their ability to deliver agents with precision [9]. Additionally, specific microbial communities in the gut ecosystem have been identified to regulate CNS tumor occurrence and progression through metabolic and immune pathways, which are referred to as the “gut–brain axis” [10].

Therefore, the present microbe-based therapeutic strategies for treating brain neoplasms are characterized by three principal modalities [11], including the direct inoculation of bacteria into the tumor milieu to achieve targeted cytotoxicity, the exploitation of bacteria’s intrinsic tropism for hypoxic tumor niches to facilitate the delivery of therapeutic compounds, thereby inducing indirect tumor cell demise, and the manipulation of the gut microbiota to enhance the efficacy of brain tumor therapies. This review discusses the critical roles of bacteria in the treatment of CNS tumors from the three perspectives mentioned above and proposes potential future directions.

## 2. Bacterium-Mediated Anti-Tumor Mechanisms and Their Application in CNS Tumors

Bacteria primarily exert their anti-tumor activity in CNS tumors by indirectly modulating the immune system, as confirmed in various tumor models [12]. Researchers have made extensive efforts to introduce attenuated bacteria or their components into the site of CNS tumors [13] to evaluate their anti-tumor effects and explore novel opportunities for brain tumor patients.

### 2.1. Mechanisms Underpinning Bacterial Anti-Tumor Efficacy

Bacteria exert their antineoplastic influence predominantly through the modulation of tumor-associated immune processes. The antigens and metabolites derived from these microorganisms can activate immune cells, thereby enhancing the body’s immune surveillance against neoplastic growth. Additionally, the fine-tuning of bacterial diversity is a critical component in the repertoire of bacterial-mediated antineoplastic activities, offering a nuanced approach to cancer therapy.

#### 2.1.1. Bacterial Activation of Immune Cells for Anti-Tumor Response

Bacteria are instrumental in fine-tuning the host’s anti-tumor immune response by directly activating immune cells and indirectly influencing the metabolic landscape of the tumor microenvironment (TME). For instance, *Bifidobacterium* species have been shown to enhance the immune response by increasing the populations of CD4+ T cells, CD8+ T cells, and natural killer (NK) cells. This enhancement is further characterized by increased production of interferon-gamma (IFN-γ) and interleukin-2 (IL-2) within the TME, concurrent with downregulation of tumor necrosis factor-alpha (TNF-α) and interleukin-10 (IL-10), thereby fortifying the host’s anti-tumor defenses [14]. In a parallel study, the bacterium *Helicobacter hepaticus* (Hhep) was found to amplify the anti-tumor immune response by inducing the proliferation of Hhep-specific T follicular helper (Tfh) cells and fostering the development of tertiary lymphoid structures (TLSs), which in turn suppresses tumor growth [15]. Beyond activating immune cells, bacteria also alter the TME’s metabolic profile, thereby sculpting the host’s immune response and the trajectory of tumor progression. Notably, certain gut commensal bacteria, such as *Ruminococcus gnavus* and *Blautia producta*, can restore the immune surveillance capabilities of CD8+ T cells by degrading lyso-glycerophospholipids within the TME [16], indirectly bolstering the host’s anti-tumor immune response through metabolic intervention.

#### 2.1.2. Bacterial Production of Metabolites for Tumor Suppression

The metabolites produced by bacteria emerge as important contributors to tumor suppression. Studies have uncovered that certain bacterial peptides can be presented by the human leukocyte antigen (HLA) molecules on glioblastoma cells, effectively engaging tumor-infiltrating T cells (TILs) and peripheral blood memory cells in the specific recognition and targeting of tumor cells [17]. Moreover, bacteria are capable of generating cyclic di-AMP, a potent stimulator of the interferon gene, which in turn activates monocytes within the tumor to produce type I interferons (IFNs). This cascade enhances the activation and functionality of immune cells, including NK cells and dendritic cells (DCs), thereby amplifying the body’s anti-tumor immune response [18]. In a groundbreaking discovery, Kovács P et al. identified that lithocholic acid, a bacterial metabolite, activates Takeda G protein-coupled receptor 5 (TGR5) and constitutive androstane receptor (CAR) by modulating the expression of nuclear factor-2 (NRF2) and Kelch-like ECH associating protein 1 (KEAP1). This activation leads to increased oxidative stress in tumor cells, which can suppress tumorigenesis [19]. Collectively, these findings underscore the multifaceted and profound impact of bacterial production of metabolites on the inhibition of tumor occurrence and progression.

#### 2.1.3. The Anti-Tumor Effects of Changes in Bacterial Diversity

Bacteria, as integral constituents of microbial communities within host tissues, can profoundly influence the immune response against tumors through shifts in their composition. Research has consistently shown that augmented alpha diversity within the tumor microbiota correlates with improved patient outcomes and an enhanced anti-tumor immune profile. In individuals with an adverse prognosis, the tumor microbiota is often dominated by *Clostridium* and *Enterobacteriaceae*, whereas a more favorable prognosis is associated with an abundance of *Proteus genus* and *Streptomyces* [20]. This suggests that the spectrum of bacterial diversity can directly modulate the immune landscape against cancer. Higher bacterial diversity within tumors has been linked to an upsurge in the presence of immune-activating CD8+ T cells and a reduction in immunosuppressive elements such as regulatory T cells and myeloid-derived suppressor cells. Furthermore, the diversity of the gut microbiota exerts a significant influence on the tumor microbiota’s composition. By strategically manipulating the gut microbiota’s diversity, it is conceivable to bolster the anti-tumor immune response, regulate the tumor microbiota’s makeup, and consequently impede tumor progression [20]. Thus, the modulation of microbiota structure emerges as a potential mechanism through which bacteria can exert their antineoplastic effects.

To date, the interplay between CNS tumors and bacteria remains an underexplored frontier in research. Brain malignancies, including gliomas, are characterized by a complex tumor microenvironment populated by a diverse array of immune cells, cytokines, and tumor cells. By drawing parallels and applying analogous research methodologies, there is potential to uncover more profound insights into the mechanisms governing these interactions.

### 2.2. Bacteria-Mediated Anti-Tumor Therapy Strategies in CNS Tumors

Recognizing the anti-tumor properties of bacteria, researchers have dedicated considerable effort to integrating attenuated bacteria and their components into the CNS tumor microenvironment. This approach aims to explore their potential for both directly killing tumors and indirectly facilitating tumor elimination by activating immune cells. These efforts involve the strategic injection of engineered bacteria or bacterial fractions.

#### 2.2.1. Attenuated Bacteria for CNS Tumor Treatment

*S.t-DpGFlaB* is an engineered *Salmonella typhimurium* with the expression of heterologous Vibrio vulnificus flagellin B (FlaB) and the inability to synthesize guanosine 50-diphosphate-30-diphosphate (*DppGpp S*) [21,22]. The absence of *DppGpp S* inhibited endotoxin expression and reduced toxicity. The FlaB protein can recruit neutrophils and repolarize M2 macrophages into M1 macrophages [23]. Mi Z et al. found the injected *S.t-DpGFlaB* could bind to the blood–brain barrier (BBB) endothelial cell line GRP94 with the outer membrane protein A (OmpA), transit the BBB, and actively target the glioma in mice [8]. Moreover, *S.t-DpGFlaB* could reshape the immune microenvironment in glioma by releasing the FlaB proteins, recruiting immune cells, and repolarizing macrophages [13]. Huang J et al. employed precise genetic engineering techniques to modify the expression of diaminopimelic acid (DAP), an essential protein synthesized in the cell wall of *Salmonella typhimurium*, producing another strain named *Salmonella YB1* [24]. In this new strain, the gene responsible for DAP expression is under the control of the *pepT* promoter, which is activated under hypoxic conditions. This regulation ensures that DAP is expressed exclusively in hypoxic environments, thereby promoting the proliferation of *Salmonella typhimurium* within the hypoxic tumor microenvironment while avoiding toxicity in oxygen-rich tissues [25]. This characteristic enables *Salmonella YB1* to effectively target tumors under hypoxic conditions and selectively grow at these sites, as demonstrated by its proven efficacy against breast cancer [24].In a study involving mice harboring human glioblastoma U87 MG, intravenous administration of *Salmonella YB1* was observed to significantly inhibit glioblastoma growth and showed a lower incidence of systemic toxicity in the tumor-bearing mice [25]. Simultaneously, *Salmonella YB1* [24] was reported to decrease glutathione peroxidase-4 expression, induce mitochondrial atrophy, and increase malondialdehyde and ROS production in glioma cells, leading to iron death in glioma cells [25]. Moreover, the growth-inhibitory effect induced by *Salmonella YB1* can be prominently counteracted by the iron death inhibitor Fer-1, providing further evidence that it induces glioblastoma cell growth inhibition, at least partially, via the iron death mechanism. This discovery opens up new avenues for research, suggesting that synergistic anti-tumor effects could be realized by combining *Salmonella YB1* with agents like the iron death inducer Erastin or other therapeutic approaches that facilitate iron death, such as sorafenib and photodynamic therapy. Therefore, engineered bacteria, such as *Salmonella typhimurium*, can directly inhibit CNS tumors or indirectly suppress their growth by activating immune cells, showcasing broad prospects for application in the field of brain tumor treatment, especially gliomas.

#### 2.2.2. Anti-Tumor Effects of Bacterial Components

Bacterial components play a significant role in the field of eradicating brain tumors. Bacterial toxins are toxic proteins produced by bacteria that can promote the invasion of bacteria into the host, establish the ecological niche required for bacterial survival, and regulate the cell cycle, protein synthesis, and cytoskeleton [26]. The promising aspect lies in the ability of bacterial toxins to exert a lethal effect on host cells through specific invasion mechanisms and potent toxicity, making them a potential drug for targeting brain tumor cells.

Cytotoxic necrotizing factor 1 (CNF1), a bacterial virulence factor derived from *E. coli*, can activate Rho GTPases and inhibit GTP hydrolysis, leading to cytotoxicity [27]. Vannini et al. demonstrated that CNF1 can inhibit the proliferation and migration of glioblastoma cells and promote their senescence and death in vitro. Moreover, in in vivo experiments, CNF1 extended the survival of tumor-bearing mice, surpassing the survival of the Temozolomide (TMZ)-treated group [28]. Interestingly, CNF1 was also able to maintain the structure and function of peritumoral neurons [29]. For example, the physiological characteristics of peritumoral pyramidal neurons and the length of neural dendrites were improved by CNF1, preserving the function of cortical networks [29]. However, the presence of the BBB necessitates intracranial injection of CNF1, thereby restricting its broader application. Vannini et al. subsequently designed and constructed a chimeric protein, namely CTX-CNF1 [30]. Chlorotoxin (CTX) is a 36-amino acid peptide [31] derived from the venom of the scorpion *Leiurus quinquestriatus* that is capable of crossing through the BBB and targeting gliomas. This chimeric protein system, when administered systemically, achieves precise targeting of glioblastoma and utilizes CNF1 to exert its anti-tumor effects. Therefore, the CTX-CNF1 protein chimeric system is an ideal candidate for the biotherapy of CNS tumors, despite the need for further safety evaluation.

Bacteriocins synthesized by bacterial ribosomes have also been found to exhibit inhibitory effects on glioblastoma. Reports have shown that Moxidectin (MOX), a bacteriocin derived from *Streptomyces cyanogriseus*, can inhibit gliomas by activating the caspase-3/caspase-9 cascade and inhibiting cyclinD1, inducing cell apoptosis, and blocking the cell cycle at the G0/G1 phase [32]. Moreover, a recent study found that MOX promotes autophagy in glioma cells by inhibiting the AKT/mTOR signaling pathway in a dose-dependent manner [33]. The effect of MOX-induced apoptosis can be attenuated by autophagy inhibitors, demonstrating that MOX-induced autophagy can also increase apoptosis. Another bacteriocin, ivermectin, which has a similar structure to MOX, also has anti-tumor effects. Researchers reported that ivermectin promotes apoptosis in human brain microvascular endothelial cells to inhibit tumor angiogenesis, inhibits mitochondrial respiration to induce mitochondrial dysfunction, and promotes oxidative stress to inhibit glioma growth in vitro and in vivo [34]. Similarly, doramectin, which belongs to the avermectin family along with ivermectin, can trigger the caspase cascade via the endogenous apoptotic pathway and induce mitochondrial damage to generate ROS, which further induces cell necrosis via the RIPK1/RIPK3/MLKL signaling axis [35]. In conclusion, bacteriocins tend to inhibit glioma growth through multiple pathways, including apoptosis, autophagy, and necrosis, which shows great potential in anti-tumor applications.

Given the evidence, the use of attenuated bacterial strains and their derivatives shows great promise in cancer therapy, especially for brain tumors. These agents have been shown to elicit a robust anti-tumor response by activating key cellular processes such as apoptosis, autophagy, and ferroptosis. Despite these promising findings, additional preclinical studies must be conducted to thoroughly assess the safety profile of such innovative therapeutic approaches.

## 3. Bacterium-Mediated Drug Delivery in the Treatment of CNS Tumors

### 3.1. Advantages of Bacteria and Bacterial Components as Delivery Vectors

#### 3.1.1. Bacterial Capacity for Penetrating the BBB

A key advantage of employing bacteria as drug carriers is their inherent capacity to penetrate the BBB, an obstacle that frequently hinders the delivery of conventional pharmaceuticals to CNS tumor sites. Recent studies have elucidated various pathways through which bacteria and their constituents can penetrate the BBB, including intracellular transport, paracellular diffusion, and co-transport mechanisms [36,37]. For instance, *Neisseria meningitidis* has been shown to harness the Opca protein to engage with fibronectin on brain microvascular endothelial cells (BMECs), thereby gaining entry into the brain [38]. Similarly, *Escherichia coli* can exploit the membrane protein GRP94 on BMECs, utilizing the paracellular route to cross the BBB [39]. A particularly intriguing bacterial component with BBB-crossing potential is the outer membrane vesicle (OMV). These vesicles, containing lipopolysaccharide (LPS), are specifically recognized by toll-like receptors on neutrophils [40], leading to their engulfment. The neutrophil–OMV complex then actively navigates toward the endothelial junctions, employing a series of rolling, adhesion, and crawling movements, which ultimately enable their passage across the BBB [41]. Consequently, the use of bacteria and their components as drug carriers presents a promising avenue for the targeted delivery of therapeutics to the brain, circumventing the impediments posed by the BBB and offering new hope for the treatment of CNS malignancies.

#### 3.1.2. Harnessing the Precision of TME Targeting

Bacteria possess an extraordinary ability to selectively target the immunosuppressive microenvironment of tumors [42]. This targeting capability is particularly evident in certain anaerobic bacteria and their derivatives. For instance, facultative anaerobic bacteria like *Salmonella typhimurium* strain *VNP20009* and *Salmonella typhimurium* exhibit a preference for the hypoxic core of tumors, enabling them to selectively colonize the tumor microenvironment following systemic injection [43,44]. Similarly, anaerobic *C. novyi NT* spores actively seek out the hypoxic environment within tumors [45]. Furthermore, *Salmonella typhimurium A1* has been observed to be confined to necrotic areas of the tumor, where abundant nutrients support bacterial growth and proliferation [46]. Moreover, the immunosuppressive microenvironment of tumors, which is characterized as an immune-privileged site, protects bacteria against immune cell clearance, a process that occurs in normal tissues. This feature endows bacteria with enhanced specificity and targeting capabilities within the tumor microenvironment [47]. In the context of glioblastoma tumors, the hypoxic and immunosuppressive regions, typically located in the highly tumorigenic core, play a crucial role [48]. Therefore, bacteria-mediated targeted therapy holds immense potential for combating malignant brain tumors [47].

#### 3.1.3. Penetration of Bacteria into the Deep Regions of Tumors

The motility conferred by bacterial flagella facilitates the penetration of bacterial vectors into the neoplastic microenvironment, allowing access to quiescent cell populations within tumors that are often refractory to traditional chemotherapeutic agents [49]. This innate capability of bacteria presents a promising route for the targeted delivery of therapeutics to the core regions of solid tumors, where conventional treatments frequently fall short. Intriguingly, bacterial flagella not only enhance the penetration of therapeutics into the tumor’s interior but also adeptly capture tumor antigens, ferrying them to the periphery. This crucial action triggers the activation and maturation of DCs, subsequently bolstering immune responses specific to the tumor antigens [50], thus opening new avenues for immunotherapeutic strategies.

#### 3.1.4. Biocompatibility of Attenuated Bacteria

Although bacteria are known to provoke adverse immune reactions, their potential as therapeutic agents is enhanced through the precise inactivation or genetic excision of virulence factors, thereby neutralizing their toxicity [51]. Furthermore, the employment of indigenous, symbiotic bacteria such as *Bifidobacterium bifidum* [52] offers a novel approach to compound delivery. In this context, the utilization of attenuated, non-pathogenic bacteria emerges as a viable biocompatible platform for drug delivery, expanding the horizons of microbial-based therapeutics.

#### 3.1.5. Genetic Programmability and Modifiability by Biological and Chemical Methods of Bacteria

The benefits of using bacteria as drug carriers arise from their versatility, including the application of diverse techniques such as genetic engineering, bioconjugation, utilization of bacterial outer membrane vesicles, and encapsulation through bacterial ghost technology [53,54,55]. First, bacteria can be genetically engineered through plasmid introduction to express specific anti-tumor peptides and proteins, enabling them to bind as ligand-specific receptors overexpressed on tumor surfaces. This approach facilitates the loading of gene drugs [56]. Additionally, drugs can be bioconjugated to bacteria by chemically attaching them to the bacterial surface using covalent linkages. The presence of free thiol and amine groups in the bacterial cell wall allows for effective surface modification and drug delivery to tumor sites [57]. Moreover, the incorporation of polymers like polydopamine, which acts as a bridge between bacteria and drugs, enables drug attachment onto the bacterial surface [58]. Notably, nanoscale bacterial outer membrane vesicles and bacterial ghosts, which are inactive hollowed-out bacteria, offer alternative strategies for drug encapsulation [59]. Various approaches—including physical ultrasound methods, non-covalent binding, surface modification, self-assembly, and membrane fusion strategies—facilitate drug entry into these carriers [59,60]. Collectively, these diverse bacterial loading methods, which integrate chemistry, biology, materials science, and engineering, underscore the immense potential of bacteria as versatile and modifiable drug delivery platforms.

### 3.2. Bacteria and Their Components as Drug Delivery Platforms for the Treatment of CNS Tumors

The presence of the BBB and the tumor-specific microenvironment are the main reasons for the poor efficacy of chemotherapeutic drugs for CNS tumors. Bacteria and their components can not only target the BBB and brain tumor-specific acidic hypoxic microenvironments according to their biological features but also carry chemotherapeutic drugs, photosensitizers, or other drugs to achieve tumor-targeted delivery [61]. The following bacteria and their derivatives were used as delivery platforms: bacteria, bacterial outer membrane vesicles (OMVs), bacterial membrane proteins, bacterial spores, and bacterial toxins.

#### 3.2.1. Bacteria

**Figure 1:**
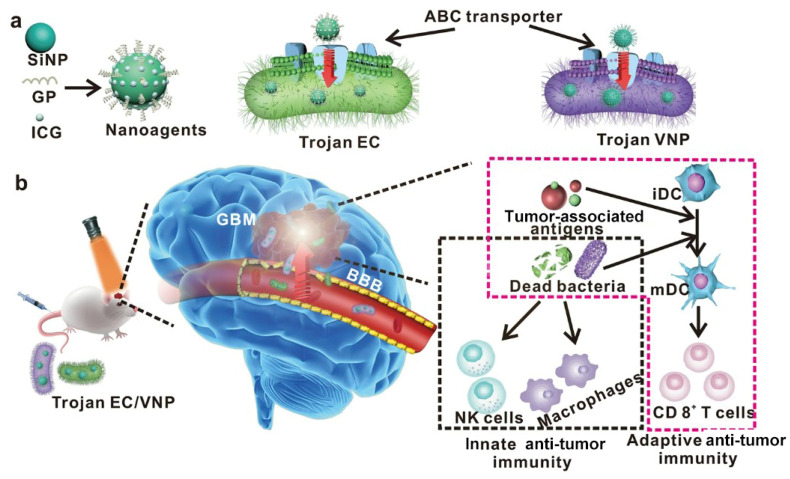
Photothermal immunotherapy of brain tumors mediated by attenuated bacteria: (**a**) bacteria form the *Trojan* Bacterial System by endocytosis of nanoparticles via the ABC transporter; (**b**) the *Trojan* Bacterial System crosses the BBB and targets brain tumors, where bacterial lysis triggers an immune response under exogenous near-infrared light irradiation. Reproduced with permission [11]. Copyright 2022, Springer Nature. Abbreviations: SiNP, silicon nanoparticle; GP, glucose polymer; ICG, indocyanine green; Trojan EC, Trojan *Escherichia coli* 25922; ABC, ATP-binding cassette; Trojan VNP, Trojan *Salmonella typhimurium* VNP20009; GBM, glioblastoma; BBB, blood–brain barrier; iDC, immature dendritic cell; mDC, mature dendritic cell; NK cells, natural killer cells.

Bacteria can be used as a drug delivery system for wrapping anti-tumor drugs to form a bacteria–drug capsule. Bacteria can pass through the BBB via phagocytosis, infiltration of endothelial cells, or the paracellular pathway [62,63]. Sun R et al. constructed a nanosystem containing glucose polymers and photosensitive indocyanine green (ICG) silicon nanoparticles (GPICG-SiNPs) [11]. GPICG-SiNPs can be endocytosed by *E. coli* and *Salmonella typhimurium* with the bacteria-specific ATP-binding cassette (ABC) transporter on the surface [64,65]. This bacteria–drug capsule system, named Trojan bacteria, can penetrate the BBB and target GBMs (Figure 1). Under irradiation with an 808 nm laser, photosensitive ICG is activated to destroy bacteria and burn the tumors. Interestingly, the antigens released from bacteria and tumors can further activate innate and adaptive immunity to promote phagocytosis and infiltration of CD8+ T cells to kill the tumors, prolonging the survival of mice with GBM in situ [11].

In addition, bacteria can also carry gene-based medicines, such as plasmids, as drug delivery vectors. Wen M et al. utilized ppGpp-deficient *Salmonella typhimurium* to deliver a plasmid-expressing tissue inhibitor of metalloproteinases 2 (TIMP-2) to gliomas. Induced by L-arabinose, *Salmonella typhimurium* was able to express TIMP-2, which can inhibit the expression of invasion-associated protein matrix metallopeptidase 2 [56]. This system can inhibit the growth of glioma cells and significantly prolong the survival of U87MG/Fluc-bearing nude mouse [66]. Attenuated *Salmonella typhimurium* could colonize, accumulate, and reproduce in the microenvironment for 14 days and is undetectable in other normal tissues, indicating that attenuated *Salmonella typhimurium* is a safe and efficient vector targeting tumors. Delivery of gene-based medicine has always been a challenge in the clinical practice of gene therapy. Attenuated bacteria may be a potential vector for gene-based drugs, such as small interfering RNA, short hairpin RNA, and antisense nucleotides. in after the specific treatments

#### 3.2.2. Outer Membrane Vesicles

OMVs secreted by bacteria are spherical carriers that possess a bilayer lipid membrane. They are of a nanometer scale and can be used as drug-carrying vehicles [67,68]. The toxicity of chemotherapeutic agents can be reduced by the encapsulation of OMVs. Moreover, neutrophils possess the innate ability to selectively recognize and phagocytose OMVs due to their bacterial antigens and pathogen-associated molecular pattern characteristics, thereby protecting the body from the toxic effects of bacterial invasion [69,70]. Mi Z et al. utilized OMVs produced by *S.t-DpGFlaB* [21] to encapsulate doxorubicin (DOX) to construct a drug delivery nanosystem, OMVs/DOX, that could target glioma [13]. OMVs are emerging as promising delivery vehicles, particularly for diseases characterized by inflammatory environments. Leveraging the power of immune cells, OMVs can home in on affected areas, thereby minimizing the systemic toxicity associated with chemotherapy.

#### 3.2.3. Bacterial Membrane Proteins

**Figure 2:**
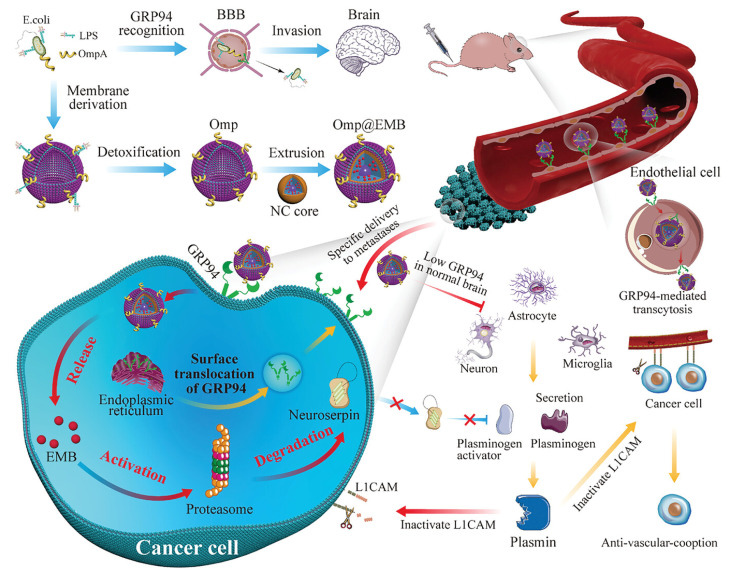
Bacterial outer membrane protein-mediated targeted drug delivery. Targeting GRP94 using attenuated *Escherichia coli* DHα5 outer membrane proteins to target penetration of the BBB as well as access to the interior of brain metastases and carry EMB to induce apoptosis in tumor cells. Reproduced with permission [72]. Copyright 2023, Wiley-VCH. Abbreviations: *E. coli*, *Escherichia coli*; GRP94, glucose-regulated protein 94; BBB, blood–brain barrier; Omp@EMB, outer membrane protein-coated Embelin; NC core, nanocapsule core; L1CAM, cell adhesion molecule L1.

Bacterial-detoxified membrane proteins, which are highly invasive, are potential candidates for drug delivery. *E. coli* can attach to the BBB endothelial cells and cross the BBB with its outer membrane protein [36], which can be a candidate for brain drug delivery carrier by the detoxification of lipopolysaccharide (LPS). Embelin (EMB) can inhibit the secretion of neuroserpin in brain metastases [71]. Zhou M et al. encapsulated EMB by detoxifying the outer membrane protein of *E. coli* (Omp@EMB) [72], which initiated cytophagy through activation of the membrane protein GRP94, facilitating the passage of the BBB, entry into brain metastatic breast cancer cells (BMBCCs), and releasing EMB (Figure 2). Moreover, this system could also restore fibrinolytic activity by inactivating the cell adhesion molecule L1 (L1CAM), which would contribute to inhibiting tumor angiogenesis and inducing apoptosis in BMBCCs [72].

**Figure 3:**
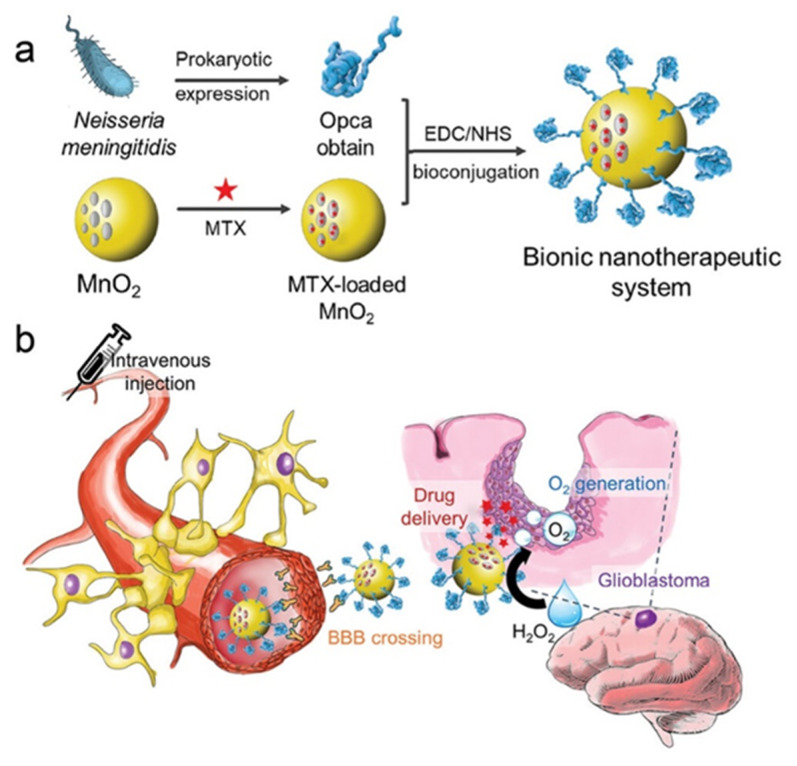
Targeted delivery of chemotherapeutic agents mediated by bacterial outer membrane proteins: (**a**) *Neisseria meningitidis* outer membrane protein Opca is combined with MnO_2_-wrapped MTX to form a bionic nanotherapeutic system; (**b**) The bionic nanosystem crosses the BBB, targets brain tumors, and uses the catalytic effect of MnO_2_ to alleviate the hypoxic environment inside the tumor and reverse the tumor’s resistance to MTX. Reproduced with permission [38]. Copyright 2022, Wiley-VCH. Abbreviations: MTX, methotrexate; MnO_2_, manganese dioxide; Opca, outer membrane invasion protein; EDC/NHS, 1-ethyl-3-(3-dimethylaminopropyl)carbodiimide/N-hydroxysuccinimide; BBB, blood–brain barrier; H_2_O_2_, hydrogen peroxide.

It has been shown that Neisseria meningitidis can cross the BBB through the Opca protein [36,73,74], which can recognize fibronectin in microvascular endothelial cells in the brain. However, the toxicity of *Neisseria meningitidis* limits its application. Researchers extracted and modified the Opca protein with MnO_2_ and subsequently wrapped it in the chemotherapeutic agent methotrexate (MTX) to form a new nanosystem, MTX@MnO_2_-Opca [38], mimicking the invasion of *Neisseria meningitidis* into the brain. This system was reported to penetrate the BBB, accumulate, and release MTX in gliomas. In addition, MnO_2_ catalyzed H_2_O_2_ in the TME, releasing O_2_ to alleviate the hypoxic environment. It also serves as a sensitizer for MTX to reverse drug resistance (Figure 3).

#### 3.2.4. Bacterial Spores

Bacterial spores are a promising drug delivery system that can induce anti-tumor immunity and target tumors. Spores are inactive bacteria that do not function in oxygen-rich areas but proliferate in anoxic environments [75,76]. Solid brain tumors contain hypoxic areas, which are immunosuppressive microenvironments that are resistant to chemotherapy and targeted therapy [77,78].

Currently, the most widely studied spore is *C. novyi NT* [79], which is motile and sensitive to hypoxia signals [80]. Researchers knocked out the α toxin gene of *C. novyi NT* to form an attenuated, spore-composed Gram-positive strain [79]. Research has shown that systematic injection of *C. novyi NT* into mice leads to spore production and extensive proliferation within the tumor, resulting in oncolysis and activation of the body’s immune response. Therefore, researchers have applied spores for the delivery of drugs [81,82,83]. Zhu L et al. coated *C. novyi NT* with the functional peptide melittin hydrogel scaffold and loaded them with metformin to form MRM-coated spore nanoparticles [84]. This nanosystem can actively target and penetrate the hypoxic and immunosuppressive microenvironments of GBM. In addition, from a mechanistic perspective, bacterial spores play the role of an “immune cells trainer” in the immune microenvironment of GBM [85], promoting DC maturation, M1 polarization of macrophages, activation of CTLs and memory T cells, as well as stimulating IFN-γ secretion, making significant contributions to innate and adaptive immune anti-tumor responses. Therefore, in targeted therapy for CNS tumors, bacteria spores act not only as the “navigations” for targeting tumors but also as the “bombs” for eliminating tumors, which show favorable prospects in the targeted therapy of CNS tumors.

#### 3.2.5. Bacterial Toxins

Bacterial toxins can serve as effective ligands for the precise targeting of tumors and the BBB, facilitating the delivery of therapeutic drugs directly to the tumor site. Cholera Toxin Subunit B (CTB) is a component of the cholera toxin deficient in virulence. The ganglioside GM1 is a glycosphingolipid expressed in gliomas and cerebrovascular endothelial cells. It also serves as an extracellular receptor that can be targeted [86]. Guan J et al. constructed a poly (lactic-co-glycolic acid)-coated nanosystem of CTB and paclitaxel (PTX) (CTB-NP/PTX), which was found to target the BBB and gliomas, ablating tumor neovascularization and releasing PTX to eliminate gliomas [87].

The engineered bacterial delivery system (EBDS) presents a promising and innovative approach for treating brain tumors, with the potential to become a future treatment option. By leveraging the unique capabilities of bacteria and their components, this system can precisely target and deliver drugs to the site of the brain tumor, offering a novel therapeutic strategy. This approach addresses certain limitations associated with conventional drugs by circumventing the BBB and selectively homing in on the brain tumor, thereby minimizing systemic toxicity and treatment resistance. However, the EBDS still faces several challenges that need to be addressed. Ensuring the safety and efficacy of the system is a paramount concern. Additionally, the development and production of this system pose technological and cost-related challenges that warrant attention. As technological advancements and clinical insights deepen, the EBDS is anticipated to significantly improve treatment outcomes for brain tumor patients, paving the way for exciting new avenues in brain tumor therapy.

## 4. Gut Bacteria and CNS Tumors

Gut bacteria mainly consist of *Firmicutes* and *Bacteroidetes phyla* [88]. They are influenced by diet, lifestyle, and drugs. Like the genetic locus, the intestinal flora of each individual is unique and easily editable. The development of fecal microbiota transplantation (FMT) and engineered bacteria has made it possible to artificially edit the intestinal flora, leading to novel therapies for various diseases. Gut bacteria communicate bi-directionally with the brain via the gut–brain axis [89], interfering with cognition [90] and influencing the development of nervous system diseases including Parkinson’s disease (PD), Alzheimer’s disease (AD) [91,92,93], and the progression of intracranial tumors [94,95]. Gut bacteria influence the progression and metastasis of intracranial tumors by regulating metabolism and TME. In this section, we summarize the mechanisms by which gut bacteria affect CNS tumors and their potential therapeutic perspectives.

### 4.1. Gut Bacteria and Tumor Progression

In the intricate dance of cellular transformation, brain tumors orchestrate a metabolic symphony that facilitates their unbridled proliferation [96]. Gut bacteria could profoundly influence the body’s metabolism and modulate the growth and invasiveness of brain tumors through the metabolic pathways [10,97,98,99,100,101], which are summarized below. We also delved into the role of bacterial exosomes in tumorgenesis and the intriguing phenomenon of reverse causality.

#### 4.1.1. Short-Chain Fatty Acids

Short-chain fatty acids (SCFAs), which include butyrate, propionate, and acetate, are volatile fatty acids with straight or branched chains of up to five or six carbons and are primarily produced by gut bacteria through the fermentation of food [102]. Firmicutes are one of the major sources of butyrate in the gut [103], while the Anaplasma phylum produces mainly propionate. SCFAs are important in the immune response, enhancing the potential of CD8+ T-cells to convert into memory cells [104] and recruiting neutrophils by modulating the cytokine CXCL1/8 [105].

Researchers reported that the proportion of Firmicutes in the gut was significantly lower in patients with malignant brain tumors compared to those with benign tumors and healthy controls, while the Anaplasmosis phylum was more abundant in patients with benign brain tumors [94,97,106]. This suggests that SCFA-producing probiotics, along with SCFAs, are valuable tools for the treatment and diagnosis of brain tumors [107]. SCFAs can penetrate the BBB, promote the activation and maturation of microglia [108], and reverse global defects in microglia in germ-free mice (GFs), suggesting that SCFAs produced by bacteria are crucial in the construction of the intracranial immune microenvironment. In addition, SCFAs, as histone deacetylase inhibitors, can reduce vascular endothelial growth factor (VEGF) secretion and decrease angiogenesis in gliomas [109]. SCFAs can inhibit the proliferation of glioma cells through up-regulation of the cell-cycle-regulating proteins p21, p27, and p53 [110]. SCFAs can also reduce the invasiveness of gliomas, despite the fact that the exact mechanism has not been elucidated [110]. SCFAs also play a role in synergistic therapy and efficacy sensitization, including synergizing with curcumin and quercetin to induce apoptosis in GBM cells [111,112], enhancing glioma sensitivity to herpes simplex virus thymidine kinase (HSV-TK)/ganciclovir gene therapy by promoting a bystander effect in glioma cells [113], and increasing the sensitivity of glioma to radiotherapy [114]. Consequently, SCFAs and SCFA-producing probiotics are potential targets for brain tumor therapies.

#### 4.1.2. Arginine

Arginine is a semi-essential amino acid derived from exogenous uptake and endogenous citrulline conversion mediated by argininosuccinate synthetase and argininosuccinate lyase. Gut bacteria produce polyamines and nitric oxide (NO) through the catabolism of arginine. Arginine metabolism is an important metabolic pathway affecting tumor progression [115]. In malignant tumors, DNA synthesis, energy metabolism, and protein synthesis are more active, and the demand for exogenous arginine is increased. As a result, malignant tumors, especially those deficient in argininosuccinate synthetase (ASS), are especially sensitive to arginine deficiency. Arginine deprivation therapy was shown to increase the sensitivity of GBM to radiotherapy [115,116]. Therefore, intestinal flora are expected to kill tumors by reducing blood arginine levels. In addition, metabolites of arginine, including polyamines and NO, can penetrate the BBB and profoundly influence the TME. Polyamines, which are highly basic compounds, promote the survival of myeloid cells and myeloid-driven immunosuppression in the acidic and hypoxic microenvironments of gliomas [117,118]. NO in the immune microenvironment can induce immunogenic cell death, normalize tumor vessels, and promote sensitivity to chemotherapeutic agents [119,120,121,122,123]. Therefore, the strategic modulation of arginine metabolism by gut bacteria emerges as a promising avenue for the suppression of tumor growth.

#### 4.1.3. Tryptophan

Tryptophan, an indispensable amino acid, exists in two forms: it can be found free or complexed with albumin, and it is the free form that can traverse the BBB [124]. *Firmicutes*, *Anaplasma*, *Actinobacteria*, *Clostridia*, and *Aspergillus* are involved in tryptophan metabolism and produce a range of metabolites, such as kynurenine, 3-Hydroxykynurenine, 3-Hydroxyanthranilic acid, alpha-amino-beta-carboxy-muconate-epsilon-semialdehyde, quinolinic acid, and aminomuconic semialdehyde [125,126]. Free tryptophan and its derivatives can participate in TME immunoregulation and tumor proliferation, suggesting that gut bacteria could affect TME through tryptophan metabolism. In glioma, tryptophan is catabolized to kynurenine by tryptamine 2,3-dioxygenase (TDO), after which kynurenine is further converted to quinolinic acid. The level of TDO is positively correlated with the proliferation index of brain tumors, suggesting that tryptophan metabolism mediates tumor progression [127]. Aryl hydrocarbon receptor binding with kynurenine catalyzed by TDO could inhibit anti-tumor immune effects and reduce the infiltration of CD8+ immune cells, increasing the invasiveness of gliomas. Quinolinic acid is another derivative of tryptophan produced by gut bacteria. Despite its inability to cross the BBB, quinolinic acid has been found to accumulate in gliomas [126]. Quinolinic acid affects the TME of gliomas by acting on N-Methyl-D-aspartic acid (NMDA) receptors and the forkhead box O1 (Foxo1)/peroxisome proliferator-activated receptor γ (PPARγ) signaling pathway, inducing a tumor-supportive phenotype in macrophages. The manipulation of tryptophan and its metabolic derivatives presents a compelling target for the development of therapeutic strategies against brain tumors.

#### 4.1.4. Glutamate

The primary source of glutamate (Glu), a crucial neurotransmitter, is dietary intake. Glu and its metabolic derivatives, such as α-ketoglutarate, have been implicated in the complex process of brain tumorigenesis. α-ketoglutarate can be converted from Glu with the regulation of bacilli in the gut [128]. The production of α-ketoglutarate induces DNA methylation, while the DNA methylation of key genes, for example, isocitrate dehydrogenase (IDH)1/2, leads to dysregulation of the epigenetic status of tumors, promoting brain tumors [96]. These insights pave the way for innovative therapeutic strategies that leverage the gut–brain axis, targeting Glu metabolism within the gut microbiome as a novel approach to combating brain tumors.

#### 4.1.5. Lactate

Lactate is a byproduct of aerobic glycolysis. In the gut, it is predominantly produced by lactic acid bacteria (LAB), which are part of the transient gut microbiome derived from food sources [129]. In glioma, a combination of *Lactobacillus plantarum* and *Bifidobacterium bifidum* has been shown to inhibit glioma growth by suppressing the phosphoinositide 3-kinase/serine-threonine kinase (PI3K/AKT) pathway [130]. This suggests that LAB-based microbial formulations or their metabolic byproducts, such as lactate, could be potential therapeutic options for glioma treatment [131]. However, some researchers indicated that lactate accumulated in gliomas could induce regional metabolic reprogramming and activate oxidative metabolism to protect GBM cells from nutrient deprivation-mediated cell death [132,133]. The contradictory roles of lactate in glioma growth currently lack a clear explanation, highlighting the need for further research to elucidate the impact of gut microbiota and lactate on the development of brain tumors.

### 4.2. Gut Bacteria and Tumor Therapy (Table 1)

#### 4.2.1. Chemotherapeutic Drugs Can Promote a Balanced Intestinal Flora

TMZ, a first-line chemotherapeutic agent for glioma, is traditionally believed to induce alkylated DNA lesions to combat tumors. However, emerging studies [10,95] suggest that TMZ can also ameliorate imbalances in the intestinal flora caused by gliomas. These restorative effects include increased diversity, the resurgence of *Firmicutes*, and alterations in the metabolism of amino acids, oligopeptides, bile acids, and SCFAs in the gut. Nevertheless, the current study did not directly elucidate whether the impact of TMZ on the intestinal flora of glioma patients synergistically influences its efficacy, which represents a potential direction for further investigation. It is plausible that TMZ could facilitate the activation and maturation of intracranial microglial cells by inducing the reestablishment of subpopulations, such as *Firmicutes*, in the intestinal flora. An increased abundance of Firmicutes leads to increased production of metabolites, such as SCFAs, thereby combating tumors [103].

**Table 1:** Examples of clinical trials in gut microbiota and cancer clinical trials on the role of gut microbiota in cancer therapy.

Main ID	Malignancy	Objective	Intervention	Study Type
NCT05373381	High-grade glioma	To evaluate adherence to sHFLC + KetoPhyt diet and changes in gut microbiota	sHFLC + KetoPhyt diet	single arm, unblinded
NCT03278249	glioma	To evaluate the efficacy of the ketogenic diet	Modified Atkins Ketogenic Diet	single arm, unblinded
NCT02939378	recurrent glioblastoma	To evaluate the efficacy and safety of ketogenic diet adjuvant to CRT	Ketogenic Diet	parallel assignment, open label
NCT02302235	glioblastoma	To evaluate the efficacy and safety of ketogenic diet adjuvant to radiotherapy and TMZ	Ketogenic Diet	single arm, unblinded
NCT03838601	Locoregionally advanced oropharyngeal squamous cell carcinoma	To evaluate the safety, tolerability, and engraftment of combination therapy	MET-4 plus CRT	single arm, unblinded
NCT04264975	solid cancer	To evaluate the effect of FMT	FMT	single arm, unblinded
NCT06039644	breast cancer	To evaluate the effect of probiotics on meliorating the side effects of chemotherapy in breast cancer	Probiotic	double-blind, randomized, controlled trial

Abbreviations: CRT, chemoradiotherapy; FMT, fecal microbiota transplantation; MET-4, microbial ecosystem therapeutics; sHFLC, supplemental high-fat low carbohydrate; TMZ, temozolomide. Data from https://clinicaltrials.gov/.

#### 4.2.2. Gut Bacteria Can Enhance Sensitivity to Therapy

The gut microbiota has a substantial impact on the development of tumors. It can either promote or suppress tumor growth through a range of metabolic functions and interactions with immune factors and cells. Currently, one of the major challenges in the application of chemotherapeutic drugs lies in understanding the varying effects of these drugs on different subtypes of the same tumor cells. Determining which molecules or characteristics are closely associated with drug sensitivity is crucial for enhancing the efficacy of existing chemotherapy. Interestingly, several studies, particularly those focused on intracranial tumors, have uncovered the previously underestimated influence of intestinal flora on tumor sensitivity to drugs. These studies demonstrated a notable difference in the β-diversity of intestinal flora between the TMZ-sensitized and non-sensitized groups. The non-sensitized group exhibited a higher abundance of Bacteroides, *Alloprevotella*, *Muribaculum*, and *Desulfovibrio*, while the sensitized group had a higher abundance of *Akkermansia* [10]. These two groups differed significantly in steroid and tryptophan metabolic pathways, as well as in macrophage and cytotoxic T lymphocyte infiltration [10]. Previous research has indicated that broad-spectrum antibiotic cocktail (ABX) therapy can lead to tumor-promoting effects by reducing cytotoxic NK cell subsets and impairing microglial function in the intracranial tumor microenvironment [100,134]. These findings are consistent with the results of the aforementioned studies, highlighting the crucial role of the intestinal flora as a target for sensitizing chemotherapeutic agents. The modulation of the intracranial immune microenvironment by the intestinal flora appears to mediate anti-tumor effects and influence chemotherapeutic drug sensitivity.

#### 4.2.3. Targeting the Intestinal Flora as a Novel Approach for Intracranial Tumor Therapy

##### Fecal Microbiota Transplantation

FMT has emerged as a crucial validation approach in animal experiments, as it has the potential to inhibit tumor development, mitigate resistance to chemotherapeutic drugs, and alleviate adverse events. Several ongoing clinical trials focus on FMT as a treatment for intracranial tumors or as a strategy to reduce drug toxicity. An illustrative example is the ability of FMT to reverse melanoma resistance to anti-PD-1 therapy [135]. Given that the intestinal flora comprises a complex network of interconnected bacteria, FMT represents a simpler method for modifying the patient’s intestinal flora compared to approaches involving phage therapy, genetic modification, or small molecules. Consequently, FMT holds promise as one of the earliest therapeutic interventions applied in clinical settings to target the intestinal flora in tumor patients.

##### Probiotics

Oral probiotics represent an alternative approach to modifying the intestinal flora. Based on previous research highlighting the ability of intestinal flora to impede the growth of intracranial tumors, probiotics hold great promise as an ideal method for modifying intestinal flora [136,137]. Currently, there are ongoing clinical studies investigating the use of probiotics to modify the intestinal flora. For instance, Ashley A. Hibberd et al. observed an increase in butyrate-producing flora in the feces, tumor mucosa, and non-tumor mucosa following oral probiotics given to patients with colon cancer. This finding suggests that utilizing probiotics to improve the composition of the intestinal flora in tumor patients is effective [138]. However, this study did not further substantiate the role of probiotics in prolonging the survival of patients with tumors. Another study demonstrated that the combination of oral *Lactobacillus casei* with epirubicin prolonged recurrence-free survival in bladder cancer patients [139]. In conclusion, probiotics are prospectively to be applied in clinical practice to modulate the intestinal flora in oncological patients.

##### Diet

The composition of the intestinal flora is influenced by diets [136,140], leading to individual variability, which poses challenges in studying intestinal flora consistently across different illnesses. Interestingly, obese mice have an increased ratio of *Firmicutes* to *Bacteroides* in their intestinal flora [141]. In patients with intracranial malignant tumors, the abundance of *Firmicutes* was significantly lower than that in patients with benign tumors and in the healthy population [94]. Treatment with TMZ restored the abundance of *Firmicutes* in the intestines of patients with gliomas [10,95]. These findings raise the possibility that obesity might unexpectedly serve as a protective factor for glioma patients. However, this inference contradicts the findings of several existing studies, such as the work of Tze et al., who stated that obesity is a risk factor for hepatocellular carcinoma. Therefore, additional direct evidence is needed to understand the effects of obesity on intracranial tumors. Dietary fiber may also have a protective effect on patients with intracranial tumors. Studies have shown that dietary fiber improves progression-free survival in melanoma patients [142]. Moreover, a high-fiber diet promotes the growth of bacteria that produce SCFAs [143]. These SCFA-producing bacteria play a role in intracranial glioma eradication through the activation of microglia and other pathways. A ketogenic diet could increase *Akkermansia* and *parabobacteriodes* to remodel the gut microbiota [144], which explains its effects on reducing glioma growth [145]. Dietary intervention stands as a compelling strategy to reshape the intestinal flora, thereby harnessing its potential influence on tumor therapy.

## 5. Concluding Remarks and Prospects

CNS tumors present formidable clinical challenges with a dearth of effective therapeutic strategies. The advent of bacteriotherapy, however, has sparked a surge of interest due to its promising outcomes in preclinical studies for oncological applications. This review synthesizes the burgeoning field of bacterial-mediated therapy for CNS tumors, focusing on three perspectives: the direct oncolytic action of bacteria, the use of bacteria as vectors for targeted delivery of therapeutic agents to the TME, and the strategic modulation of the gut microbiota to influence the metabolic and immunological milieu of CNS tumors, thereby exerting a suppressive effect on tumor progression.

The domain of CNS tumor therapy is currently dominated by preclinical explorations, with bacteriotherapy emerging as a novel approach. Inspired by the bactericidal properties observed in non-CNS malignancies, investigators have endeavored to harness bacteria to directly target and destroy CNS tumor cells. Yet, the cytotoxicity of bacteria alone falls short of the threshold for complete tumor eradication, highlighting the need for a synergistic approach with additional therapeutic agents at the tumor site. The BBB poses a formidable barrier, restricting the ingress of numerous compounds into the CNS tumor milieu. Nonetheless, select bacteria have demonstrated an innate capacity to breach the BBB and penetrate into the core of tumors, positioning them as potential vectors for drug delivery. Concurrently, the modulation of the gut microbiota offers a pathway to regulate the immune and metabolic landscape of brain tumors, presenting a new frontier in therapeutic intervention. Despite the promise, the clinical translation of bacteriotherapy for CNS tumors is fraught with challenges, with safety paramount among them. Although bacteria are engineered to be non-virulent and inactivated physically, rigorous animal testing and preclinical trials are essential to ascertaining their safety before human application. Moreover, the enrollment criteria for clinical trials involving intestinal flora often overlook critical variables such as diet and lifestyle, which could compromise the integrity of the research findings. Thus, the development of refined inclusion and exclusion criteria is critical for steering future investigations and trials. It is worth noting that the intratumoral microbiome of brain tumors represents an emerging field in bacterial therapy for CNS tumors. By employing techniques such as metagenomics, clarifying the significance of the intratumoral microbiome in the temporal and spatial progression of CNS tumors is crucial for the development of subsequent therapeutic strategies. The key objective is to enhance patient care and prognosis. The burgeoning role of bacteria in brain tumor research opens vast avenues for scientific discovery, with the potential to revolutionize clinical management standards for CNS tumor therapy.

## Data Availability

The data presented in this study are available in the review.
